# Clinical Features of Gollop-Wolfgang Complex in North Africa: A Case Study

**DOI:** 10.7759/cureus.87871

**Published:** 2025-07-14

**Authors:** Abderrahim Lachhab, Mohamed Maroc, Yassine Benghali, Ahmed Amine EL Oumri

**Affiliations:** 1 Faculty of Medicine, Mohammed First University, Oujda, MAR; 2 Physical Medicine and Rehabilitation, Mohammed VI University Hospital, Oujda, MAR

**Keywords:** femoral bifurcation, gollop-wolfgang complex (gwc), knee disarticulation, prenatal detection, tibial aplasia

## Abstract

Gollop-Wolfgang Complex (GWC) is a rare congenital musculoskeletal anomaly marked by distal femoral duplication and tibial aplasia. While often linked with other systemic defects like those in the VACTERL association, our case uniquely presents an isolated manifestation of this complex. The exact genetic cause of GWC isn't fully understood, highlighting a gap in our knowledge of limb development disorders. Treatment typically involves early surgical intervention, such as knee disarticulation and prosthetic fitting, though limb salvage procedures are also recognized. Despite its global rarity (fewer than 200 reported cases), GWC is rarely documented in Africa. This report details a case of GWC from North Africa, offering insights into its presentation and management within this demographic.

We present a 14-year-old Moroccan male, the third of three siblings, who presented with a right lower limb deformity evident since birth. Clinically, he showed a characteristic Y-shaped distal thigh due to palpable femoral bifurcation, a fixed knee flexion deformity, and apparent absence of the tibia. Radiographs confirmed a bifurcated right distal femur and right tibial hemimelia (Jones Type Ia). Notably, our patient had no associated upper limb, cardiac, neurological, or renal deformities, nor ectrodactyly or absent radii, distinguishing his presentation from many reported cases. Prenatal diagnosis wasn't established due to a lack of antenatal ultrasound follow-up.

Despite thorough counseling on surgical options, including amputation for prosthetic fitting, the patient declined intervention due to fears of postoperative pain and complications. Consequently, we initiated a conservative management plan focused on rehabilitation, crutch use, unipodal balance exercises, and gait training to optimize his functional independence.

This case report underscores the diagnostic challenges of GWC and highlights the critical role of patient autonomy in treatment decisions, particularly when conventional surgical approaches are met with patient refusal. Our experience suggests that a dedicated, non-surgical rehabilitation pathway can be a viable alternative, even in complex skeletal anomalies. This unique case contributes valuable clinical data, expanding the limited global understanding of GWC and emphasizing the need for comprehensive documentation of rare conditions to refine personalized management strategies.

## Introduction

Gollop-Wolfgang Complex (GWC) is a rare congenital condition affecting the musculoskeletal system. It is characterized by a duplicated distal femur and tibial aplasia, which may or may not be accompanied by ectrodactyly of the hand or foot [1,2]. GWC frequently presents alongside other congenital anomalies, often aligning with the VACTERL association. This encompasses a spectrum of birth defects, including vertebral anomalies, anal atresia, cardiovascular anomalies (most commonly a ventricular septal defect), tracheoesophageal fistula, esophageal atresia, renal anomalies, and limb defects (typically radial ray defects) [3]. The embryology of limb development is governed by intricate molecular and genetic signaling pathways, involving crucial regulatory genes and transcription factors. While the precise etiology of GWC remains unclear, it is understood to stem from an error in the complex genetic regulation of these pathways during early limb patterning [4]. Recent advances in developmental biology highlight the significant roles of genes such as HOX and pathways like Sonic Hedgehog (Shh) in proximal-distal and anterior-posterior limb axis formation, and disruptions in these mechanisms are central to understanding complex limb malformations like GWC [5,6]. While this condition can be inherited in an autosomal dominant manner with variable penetrance and expressivity, de novo mutations are also a possibility [7].

Current treatment approaches for GWC often involve early knee disarticulation and removal of the abnormal bifurcated femur, followed by prosthetic fitting [8]. However, limb salvage surgeries have also been performed, particularly when parental consent for amputation is absent, yielding initial outcomes comparable to those of amputation [9]. The initial description of this condition dates back to 1980 by Gollop et al., documenting it in two brothers with similar limb deformities. Wolfgang's report in 1984 further contributed, leading to Lurie and Ilyina coining the term GWC in 1986 [9]. GWC is exceptionally rare, with fewer than 200 cases documented globally and an estimated incidence of one in 1,000,000 live births [10].

Despite its global rarity, cases of GWC are infrequently published, especially from Sub-Saharan Africa. Our patient's presentation represents the first documented report from North Africa and the Maghreb region. Notably, despite the initial recommendation for amputation, this patient declined surgical intervention, opting instead for a rehabilitation-focused management plan.

## Case presentation

**Figure 1:**
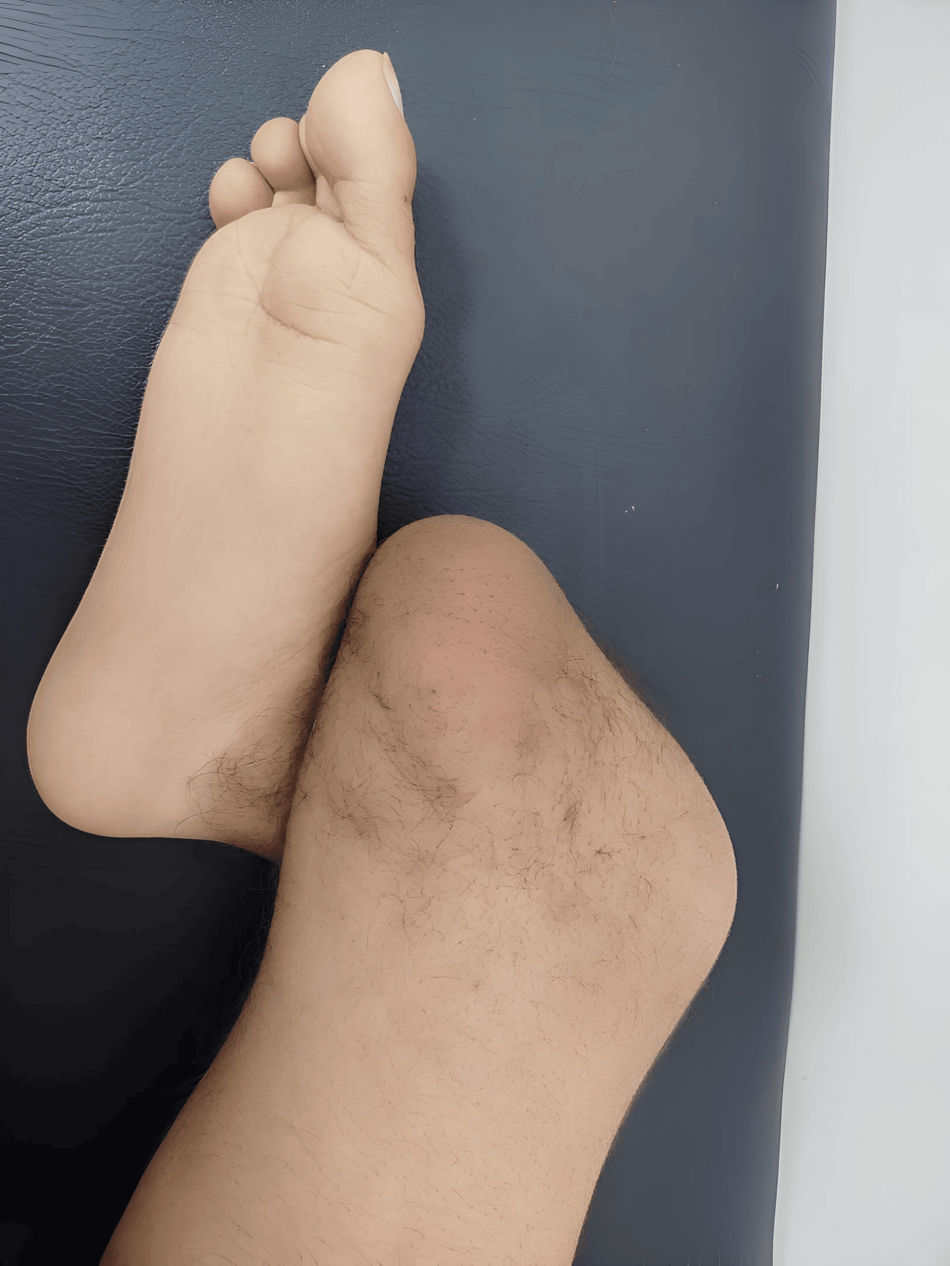
Visible deformity of the right lower limb, including shortening and the triangular configuration of the distal femur, indicative of GWC GWC: Gollop-Wolfgang complex

A 14-year-old Moroccan male, the third of three siblings with no similar musculoskeletal malformations, presented with a right lower limb deformity evident since birth. He was born at term via normal per-vaginal delivery to healthy, non-consanguineous parents who reported no significant past medical history, pregnancy pathologies, or exposure to teratogenic drugs, alcohol, smoking, or radiation. The mother did not receive regular antenatal ultrasound follow-up, which prevented prenatal diagnosis. Clinically active, well-oriented, and with normal intelligence, the patient reported no pain. A detailed clinical examination of his right lower limb revealed a widened, triangular, Y-shaped distal thigh with a palpable distal femoral bifurcation. He presented with a fixed right knee flexion deformity of approximately 100 degrees, an impalpable patella, and an inability to maintain knee extension. Limb length measurements, taken from the anterior superior iliac spine to the medial malleolus, showed the left lower limb measured 85 cm, while the affected right lower limb measured 50 cm, indicating significant shortening consistent with apparent absence of the tibia (Figure 1). He demonstrated good capillary refill and intact sensations, with normal passive and active hip movements. Neurological examination was normal, and muscle strength testing revealed grade 5/5 power for the quadriceps, plantar flexors, and dorsiflexors of the left lower limb. Initial functional assessment indicated moderate balance issues, and the patient reported occasional falls. Psychological assessment revealed an altered psychological state.

**Figure 2:**
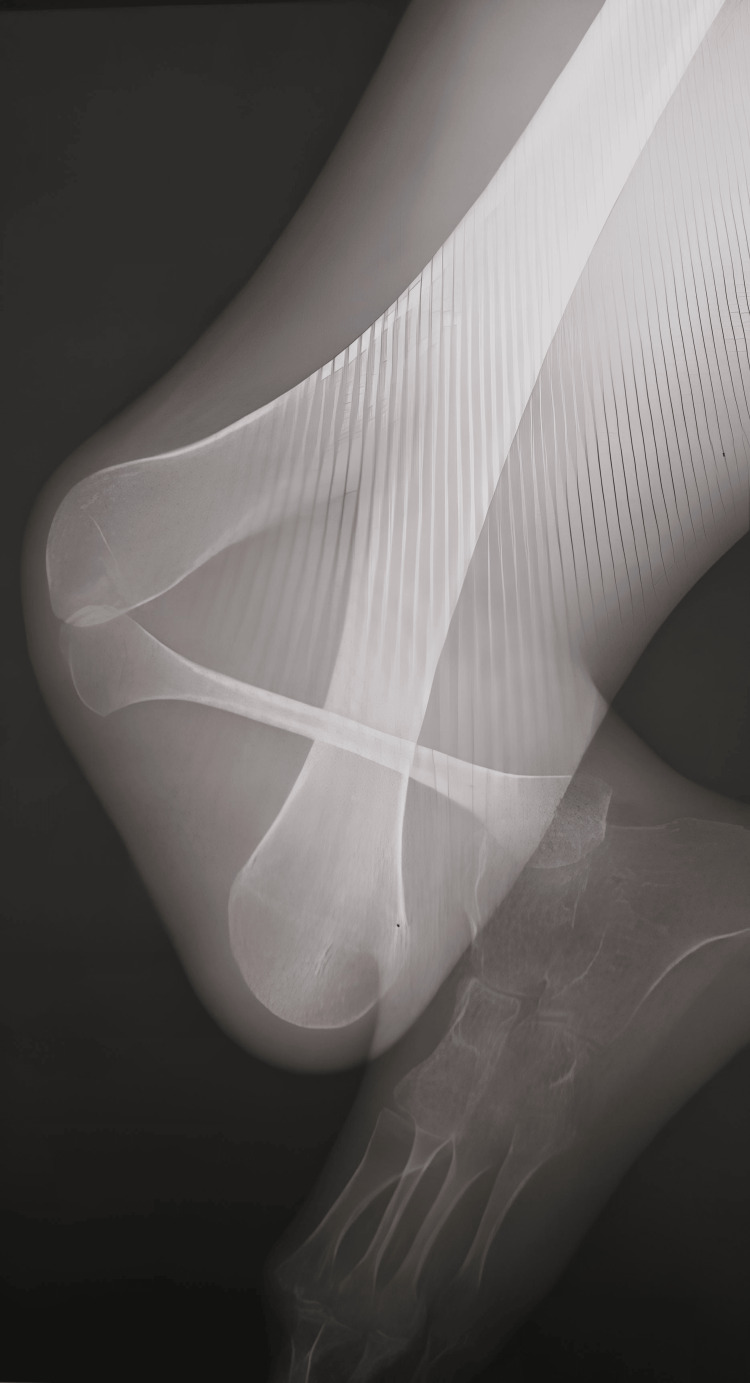
Anteroposterior radiograph of the right lower limb showing a duplicated distal femur (bifurcated femur) and complete tibial aplasia, consistent with GWC GWC: Gollop-Wolfgang complex

**Figure 3:**
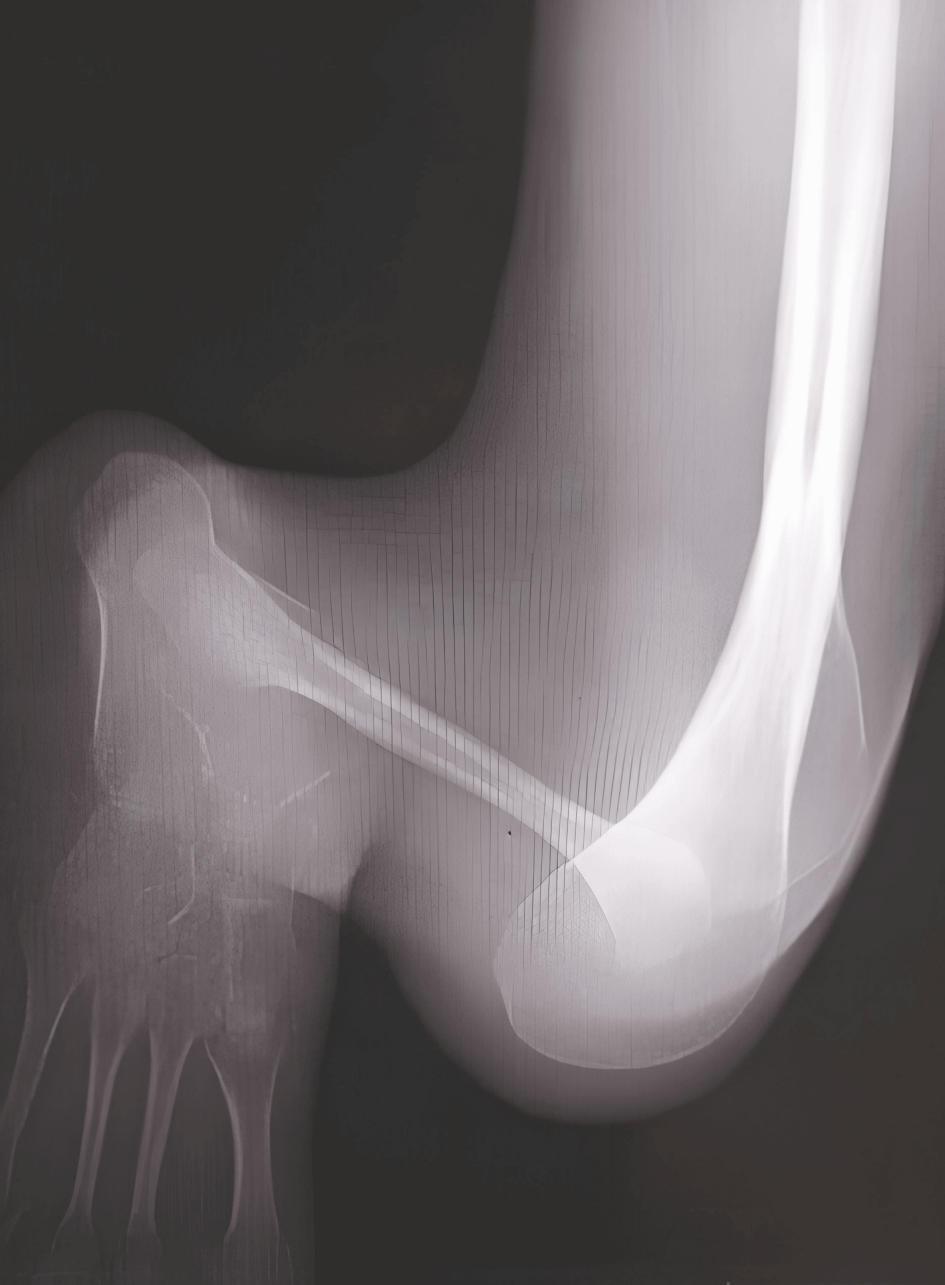
Lateral radiograph of the right lower limb demonstrating abnormal distal femoral morphology and absence of the tibia, characteristic of GWC GWC: Gollop-Wolfgang complex

Radiographic films confirmed GWC, characterized by a bifurcated right distal femur and right tibial hemimelia. The imaging specifically revealed a completely absent tibia and a hypoplastic ossific nucleus of the distal femoral epiphysis, precisely fitting the criteria for Jones Type Ia (Figures 2-3).

Further examination revealed no associated upper limb, cardiac, neurological, or renal deformities. Despite counseling regarding surgical intervention, specifically the excision of the prominent medial branch of the bifurcated femur on the right, and discussions about amputation for prosthetic fitting, the family initially favored these surgical options. However, the patient himself refused all proposed interventions, exercising his autonomy and opting solely for regular follow-up. The family demonstrated compliance with the patient's decision and the follow-up plan. Consequently, a conservative management plan was initiated, with the rationale based entirely on the patient's refusal of surgical options, prioritizing patient autonomy and focused rehabilitation. The rehabilitation was carried out by a certified physiotherapist at the hospital's rehabilitation service, consisting of 15 sessions focused on improving functional mobility, including the use of a crutch as an assistive device, unipodal balance exercises, and gait training. The Pediatric Outcomes Data Collection Instrument (PODCI) was estimated retrospectively to provide an objective measure of the patient's initial functional status; the estimated PODCI score for the Mobility and Transfers domain was approximately 30, reflecting significant functional limitations. Follow-up will continue with objective monitoring of gait and balance improvements during rehabilitation, aiming to enhance mobility and quality of life within the limitations of the patient's chosen management plan.

## Discussion

GWC is an exceptionally rare condition. The presentation in our patient, defined by specific limb abnormalities, is noteworthy due to its isolated nature [1,2]. Unlike many reported cases, where GWC occurs as part of broader systemic anomalies like VACTERL [3], our patient's isolated presentation emphasizes the spectrum of the disorder.

The etiology of GWC remains complex and is thought to involve intricate genetic regulations impacting early limb development [7]. In addressing the need for genetic interpretation, recent advancements in developmental biology highlight the significant roles of specific genes and molecular pathways in limb patterning. Mechanisms involving Shh [5] and HOX gene regulation [6] are central to understanding these complex malformations. Research has further identified specific genetic correlations, such as duplications in BHLHA9 [4] and deletions on chromosome 8q [11], offering etiological insights. While specific genetic testing was not performed for this patient, these findings are vital for interpreting the underlying mechanisms of GWC.

The management of GWC presents substantial challenges due to the lack of a standardized protocol [9], necessitating a highly individualized approach [12,13]. Historical approaches have varied, focusing on limb salvage [1,9] or knee disarticulation and prosthetic fitting [3,8]. The debate over the preferred surgical approach often balances short- and long-term functional outcomes [8]. However, our case underscores the crucial role of patient autonomy in decision-making. Comprehensive counseling was provided to the 14-year-old patient and his caregiver regarding the proposed surgical interventions, acknowledging the potential risks and benefits. This extensive consultation was essential in navigating the patient's refusal of amputation [8], emphasizing the importance of patient-centered care, particularly in pediatric cases.

Regarding the details of conservative management, the patient's fear of surgical complications led to the initiation of a conservative rehabilitation plan. The management included 15 detailed sessions focusing on gait training, unipodal balance exercises, and the use of a crutch as an assistive device. The patient and family demonstrated strong compliance throughout the process. While formal pre- and post-rehabilitation assessments using standardized scales were not performed, the initial functional status, as estimated by the PODCI mobility and transfers score of approximately 30, indicated significant functional limitations that rehabilitation aims to address.

This case also highlights critical broader implications for the management of rare congenital disorders. The lack of regular antenatal ultrasound follow-up in our patient [12] underscores the necessity of strengthening prenatal diagnostic capabilities in low-resource settings, which is crucial for early detection and counseling. Furthermore, the rarity of GWC emphasizes the importance of global registries [10] to centralize data from similar cases [14,15]. Such registries are vital for advancing research, standardizing management guidelines, and better understanding the long-term functional outcomes of both surgical and conservative approaches in these complex malformations [1,3].

## Conclusions

This case report documents a rare presentation of GWC in North Africa, highlighting an isolated limb anomaly in a patient managed without surgical intervention. This case underscores the critical importance of honoring patient autonomy in complex medical decisions, particularly in the context of a rare congenital disorder where standardized treatment protocols are lacking.

The patient’s management highlights the application of individualized, patient-centered care when surgical options are declined. Furthermore, this case emphasizes broader needs for rare congenital disorders, reinforcing the necessity of improved prenatal diagnostic capabilities in low-resource settings and the establishment of global registries to centralize data and advance comprehensive management guidelines.
